# Overlapping genes and the proteins they encode differ significantly in their sequence composition from non-overlapping genes

**DOI:** 10.1371/journal.pone.0202513

**Published:** 2018-10-19

**Authors:** Angelo Pavesi, Alberto Vianelli, Nicola Chirico, Yiming Bao, Olga Blinkova, Robert Belshaw, Andrew Firth, David Karlin

**Affiliations:** 1 Department of Chemistry, Life Sciences and Environmental Sustainability, University of Parma, Parma, Italy; 2 Department of Theoretical and Applied Sciences, University of Insubria, Varese, Italy; 3 BIG Data Center, Beijing Institute of Genomics, Chinese Academy of Sciences, Beijing, China; 4 National Center for Biotechnology Information, National Library of Medicine, National Institutes of Health, Bethesda, MD, United States of America; 5 School of Biomedical & Healthcare Sciences, Plymouth University Peninsula Schools of Medicine and Dentistry (PUPSMD), Plymouth, United Kingdom; 6 Department of Pathology, Division of Virology, University of Cambridge, Cambridge, United Kingdom; 7 Department of Zoology, University of Oxford, Oxford, United Kingdom; 8 Division of Structural Biology, University of Oxford, Oxford, United Kingdom; University of British Columbia, CANADA

## Abstract

Overlapping genes represent a fascinating evolutionary puzzle, since they encode two functionally unrelated proteins from the same DNA sequence. They originate by a mechanism of overprinting, in which point mutations in an existing frame allow the expression (the "birth") of a completely new protein from a second frame. In viruses, in which overlapping genes are abundant, these new proteins often play a critical role in infection, yet they are frequently overlooked during genome annotation. This results in erroneous interpretation of mutational studies and in a significant waste of resources. Therefore, overlapping genes need to be correctly detected, especially since they are now thought to be abundant also in eukaryotes. Developing better detection methods and conducting systematic evolutionary studies require a large, reliable benchmark dataset of known cases. We thus assembled a high-quality dataset of 80 viral overlapping genes whose expression is experimentally proven. Many of them were not present in databases. We found that overall, overlapping genes differ significantly from non-overlapping genes in their nucleotide and amino acid composition. In particular, the proteins they encode are enriched in high-degeneracy amino acids and depleted in low-degeneracy ones, which may alleviate the evolutionary constraints acting on overlapping genes. Principal component analysis revealed that the vast majority of overlapping genes follow a similar composition bias, despite their heterogeneity in length and function. Six proven mammalian overlapping genes also followed this bias. We propose that this apparently near-universal composition bias may either favour the birth of overlapping genes, or/and result from selection pressure acting on them.

## Introduction

Overlapping genes, also called “dual-coding genes”, are regions of DNA or RNA that are translated in two different reading frames to yield two different proteins. They were first detected in the genome of the bacteriophage ΦX174 [1]. For a long time they were thought to be mostly restricted to viruses, but computational [2–4] and experimental studies [5–9] indicate that they also occur in prokaryotes and eukaryotes. In particular, eukaryotic genomes probably contain numerous undetected overlapping genes, as suggested by increasing experimental evidence [10].

Overlapping genes have long fascinated biologists for three main reasons. First, they encode proteins originated *de novo* by overprinting [11, 12], as opposed to origination by well-characterized processes such as gene duplication or horizontal gene transfer [13]. Overprinting is a process in which mutations in an ancestral reading frame allow the expression of a second reading frame (the *de novo* frame), while preserving the expression of the first frame. *De novo* proteins have been little studied but are known to play an important role in viral pathogenicity [14], for instance by neutralizing the host interferon response [15, 16], the RNA interference pathway [17, 18] or by inducing apoptosis in host cells [19, 20]. In addition, those characterized so far have previously unknown 3D structural folds [22, 23] and mechanisms of action [17, 21].

The second particularly interesting feature of overlapping genes is that they represent a clear example of adaptive conflict. Indeed, they simultaneously encode two proteins whose freedom to change is constrained by each other, which would be expected to severely reduce the ability of the virus to adapt [24–30].

Finally, studying overlapping genes has greatly improved our knowledge of gene expression [31–33]. Indeed, these genes are often expressed through non-canonical mechanisms, such as transcriptional slippage [34], alternative splicing [35], leaky scanning [36], ribosomal frameshifting [37], internal ribosomal entry [38], and unconventional translation start site [39].

In spite of this interest, four decades after the discovery of overlapping genes [1], we still have only fragmentary ideas of how they originate [40], what factors favour their birth and retention [41–43], how they influence the evolution of viral genomes [12], and how they manage their inherent adaptive conflict [44].

Large-scale evolutionary studies of overlapping genes have been hampered by the limited number of known cases and by their poor annotation. On the one hand, some experimentally proven overlapping genes are not deposited in reference databases, while on the other hand, some hypothetical overlapping genes deposited in databases may be artefacts of genome annotation. In addition, overlapping genes are often overlooked during genome annotation, as proven by their recent detection in major viral pathogens of humans [45], animals [46, 47], and plants [48, 49].

Correctly detecting and annotating overlapping genes in viruses is crucial because they often encode functions necessary for *in vivo* infection [14]. For instance, a mutant phenotype initially attributed to the *Potyviridae* polyprotein is in fact due to the presence of an overlooked gene, termed *pipo*, overlapping the polyprotein frame. This overlooked gene is essential for viral replication [48]. Likewise, we must detect overlapping genes in eukaryotes because they may encode important functions and may also improve our knowledge of gene recoding [31].

To identify overlapping genes by sequence analysis, several groups have developed methods that detect the atypical pattern of nucleotide substitution induced by the overlap [50–54]. These methods detected many new potential overlapping genes in viruses [55, 56], most of which have been confirmed experimentally [45–48]. However, improvement of detection methods is hindered by the lack of a large, reliable dataset of overlapping genes, on which they could be trained and compared.

To address these issues, we asked the following questions:

- In viruses infecting eukaryotes, what overlapping genes are experimentally proven? Are some only partially proven and in need of validation? Are they all deposited in sequence databases? What is their length distribution? What are their most common mechanisms of expression? Do the proteins they encode interact with each other?
- Is the overall composition of overlapping coding regions significantly different from that of the non-overlapping coding regions? Do all overlapping coding regions follow the same pattern of sequence composition?

To answer these questions, we gathered a large dataset of experimentally proven overlapping genes from viruses infecting eukaryotes (herein called “eukaryotic viruses”). We focused on viruses for three reasons. First, they contain the vast majority of proven cases of gene overlap. Second, there is abundant information on the function and mechanism of expression of viral overlapping genes, contrary to the few eukaryotic cases known. Finally, they can inform our understanding of gene expression also in eukaryotes, since the mechanisms of gene expression used by overlapping genes from viruses infecting eukaryotic organisms can also be employed, in principle, by these organisms.

The dataset is larger and contains much more detailed biological information than previous curated datasets [12, 14, 40]. In addition, it contains both the nucleotide sequence of overlapping genes and that of non-overlapping genes in the virus genome. Statistical analysis of the dataset revealed that overlapping genes, despite their heterogeneity in length and function, share a similar composition bias.

## Materials and methods

### Assembly of a dataset of overlapping genes from eukaryotic viruses

We assembled the dataset in three steps. First, we automatically downloaded and parsed the Viral RefSeq (Reference Sequence) Release 50 file from the NCBI (National Center for Biotechnology Information) [57, 58]. It contained 2763 viral genome sequences. We extracted genes having an overlapping coding region equal to or longer than 180 nucleotides (nt). We found such genes in 894 genomes (each corresponding to a different species), corresponding to a total of 5322 overlaps. We excluded bacteriophages (174 species corresponding to 509 overlaps) and viruses with a genome longer than 30 kb (142 species corresponding to 3440 overlaps), since curation of large genomes proved too difficult [14]. Most of these genomes, indeed, have not been subject to individual review and thus are classified as “provisional” rather than “reviewed”, unlike the majority of small virus genomes [42].

Second, we excluded from the remaining 1373 overlaps 93 antiparallel overlaps (i.e. overlapping frames having an opposite orientation), which were all unproven to our knowledge. (*Note added while revising the manuscript*: 3 proven antiparallel overlaps were added by the NCBI in the RefSeq releases which followed—see Discussion). We also excluded from our automated collection 73 overlapping genes in which one or both frames were known to be interrupted by splicing events, due to uncertainties in intron-exon boundaries, which might affect the reading frame. However, we manually selected for inclusion in the dataset 3 well-characterized overlaps interrupted by splicing (the retroviral overlaps Env/Rev, Vif/Vpx, and Env/Nef), because their intron-exon boundaries can be considered reliable (they are marked with a single asterisk in S1 Table).

Third, we manually checked overlapping genes, after having selected a single representative virus species per genus. Briefly, during this step, we identified 124 independent, putatively expressed overlaps out of 1207, and discarded 1016 homologs; we also excluded 114 other overlaps, being truncated forms of overlaps already considered. During the process of manual curation (see below), we then removed 72 overlaps out of the 124 initially identified, coming to a total of 52 proven overlaps.

The initial automated analysis was performed by means of a custom-written Perl script, using 3 sources: *i*) as sketched above, the viral genomic and protein sequences based on RefSeq data extracted from the file viral.1.genomic.gbff.gz (Release 50) from NCBI website (http://www.ncbi.nlm.niv.gov); *ii*) the viral taxonomy extracted either from ICTV-Master-Species-List-2011_v2.xls (http://ictvonline.org) or from the NCBI RefSeq files if not available on ICTV; *iii*) the protein attributes extracted both from the files uniprot.sprot.dat.gz (UniProt, experimentally curated proteins) and uniprot_trembl.dat.gz (TrEMBL, computationally predicted proteins) from the UniProt Knowlegde Base Release 2011 (http://www.uniprot.org). UniProt Knowlegde Base Release 2011 (http://www.uniprot.org).

We further enriched and updated the dataset all along the curation process by adding new experimentally proven overlaps, previously misannotated or absent from the NCBI Viral Genome Database [58]. We included them into the RefSeq Viral Genome Database (see Results). Finally, before starting the present analysis, we updated each selected genome against the NCBI RefSeq viral genome database Release 80. We also updated viral taxonomy and protein attributes according to the ICTV Master Species List 2016 v1.3 and to the UniProt Knowledge Base Release 2017_12, respectively.

Overall, the curation contributed to the addition of 28 overlaps, which incorporated into the 52 which resulted from the three-step analysis described above, make the final dataset of 80 overlaps representing 61 viral species (see Results).

### Curation of experimentally proven overlapping genes

We carefully selected overlapping genes whose existence was supported by experimental evidence. For each genus, we chose only one representative overlap. To make curation useful to the community, whenever possible we chose as representative virus species that were human or plant pathogens, or that were used as model systems, or that were the type species of the genus. Often, however, evidence regarding the expression of an overlap was available only for a related species of the same genus. Not including evidence for homologous overlaps of the same genus would have considerably reduced the size of the dataset. Therefore, we decided to count as evidence of existence the experimental data available for any species in the same genus.

We classified as "reliable" proteins encoded by overlapping genes for which there was solid experimental evidence, that is proteins whose expression was confirmed using either immune detection (e.g. western blotting or immunofluorescence), or using a combination of *in vitro* translation and of observing phenotypic effects upon mutation of the overlapping frame. We classified as "to be confirmed" proteins encoded by overlapping genes for which there was partial experimental evidence (e.g. only *in vitro* translation).

In general, we report experimental evidence for the expression of both proteins from a pair of overlapping genes. In a small number of cases, the pairs of overlapping genes we collected are composed of a sequence encoding a housekeeping, phylogenetically widespread protein, and of an overlapping coding sequence discovered later. In these cases we provide experimental evidence only for the expression of the newly discovered protein.

### Detection of homologous overlapping genes

Overlapping genes can be considered homologous if they occur in phylogenetically related genera and both proteins they encode have statistically significant similarity. To ensure that overlaps of the dataset were all independent (non-homologous), we performed remote homology searches on both proteins using Psi-Blast [59] with a cut-off of significance of 10^−3^, as described [60]. First, we analyzed genomic synteny in the neighbourhood of selected overlapping genes. Whenever two pairs of overlapping genes with no apparent sequence similarity occurred in the same genome position in phylogenetically related viruses, we tested whether they were homologous by comparing the two proteins encoded by these pairs using HHpred [61] with a cut-off of significance of 10^−5^. We only kept one representative per set of homologous overlapping genes.

### Assembly of the nucleotide sequences of overlapping and non-overlapping genes

We extracted from the NCBI database the nucleotide sequence of the 80 overlapping genes that were classified as “reliable”. Their combined overall length was 35,394 nt. The 80 overlapping genes come from 61 viral genomes (the number of genomes is lower than that of overlapping genes because some genomes contain more than one overlap). We also extracted from the NCBI database the nucleotide sequence of the non-overlapping coding regions of the 61 viral genomes. In viruses with segmented genome, the non-overlapping regions were extracted from all segments. Their combined overall length was 487,158 nt.

### Comparative analysis of overlapping and non-overlapping genes

We compared the overall composition of the overlapping coding regions with that of the non-overlapping coding regions using the contingency-table chi-square test [62]. We examined five features, namely the composition in 1) nucleotides; 2) dinucleotides; 3) amino acids; 4) synonymous codons; and 5) amino acids with high codon degeneracy (the 6-fold degenerate residues L, R, and S), medium codon degeneracy (the 4- and 3-fold degenerate residues A, G, P, T, V, and I) or low codon degeneracy (the 2- and 1-fold degenerate residues C, D, E, F, H, K, N, Q, Y, M, and W).

For each composition feature in which the pooled sets of overlapping and non-overlapping coding regions differed significantly (e.g. the dinucleotide composition), we carried out a stringent chi-square test to detect what elements of the feature (e.g. what dinucleotides) were the main determinants of the difference. We used a chi-square cut-off value of 100.0 (1 degree of freedom; P<0.00001). The consistency of the composition bias revealed by the chi-square test was checked with the Wilcoxon test for paired data [63].

### Principal component analysis of overlapping genes

The chi-square analysis revealed that the overall nucleotide and amino acid composition of overlapping genes is significantly different from that of non-overlapping genes for 20 composition features (see Results). We used a multivariate statistical method, that is the principal component analysis (PCA) [64–66], to evaluate whether the observed differences were homogeneously distributed in individual overlapping genes.

We first calculated the value of the 20 composition features in each overlapping gene and in the corresponding non-overlapping counterpart. By calculating the difference between them, we obtained a matrix of 80 rows (the number of overlaps) and 20 columns. We added to the matrix an 81^th^ row, which included the difference between the 20 composition features in the pooled set of overlapping genes and those in the pooled set of non-overlapping regions. The matrix was subjected to PCA, by using the OriginPro software (OriginLab, Northampton, MA). We used the standard method, included in the OriginPro software and based on the squared Mahalanobis distance, to identify overlapping genes that are “outliers” in terms of composition.

## Results

### Collection of experimentally proven overlapping genes

We assembled a dataset of 80 overlapping genes 180 nt or longer from eukaryotic viruses with a genome shorter than 30 kb, whose expression is supported by reliable experimental evidence (see Methods). A list of the 80 overlapping genes, grouped by type of virus genome, is given in S1 Table. We also collected another 8 overlaps for which there is only partial experimental evidence (S2 Table). The 80 experimentally proven overlapping genes come from 55 genera, distributed in 30 viral families covering a wide range of viruses (Table 1). They belong to 61 virus species, 13 of them having more than one overlap.

**Table 1:** General properties of the overlapping gene dataset.

Nature of the genome	Number of families^a^	Number of genera^a^ and species (in parentheses)	Number of overlapping gene pairs^b^	Number of proteins affected by overlap^c^
**ssRNA+**	16	24 (26)	37	70
**ssRNA-**	6	12 (13)	15	29
**ssDNA**	3	9 (9)	14	26
**dsRNA**	2	5 (5)	5	10
**dsDNA**	1	1 (1)	1	2
**ssRNA-RT**	1	3 (6)	6	12
**dsDNA-RT**	1	1 (1)	2	3
**Total**	30	55 (61)	80	152

^a^Unassigned genera or unassigned families are counted as *bona fide* genera or families

^b^Some genera contain several overlapping gene pairs

^c^Some genes overlap with more than one gene

S1 Dataset contains the biological information that we collected for each pair of overlapping genes (type of experimental evidence for expression, mechanism of translation, function of the two gene products, phenotypic effects upon mutation, bibliography and other features). For each pair of overlapping genes, the file contains not only the nucleotide and amino acid sequences of the two overlapping frames but also those of the non-overlapping coding region in the virus genome. This biological and sequence information was not present in the previous curated datasets of overlapping genes [12, 14, 40]. Overall, our dataset contains 37 overlaps that were included in the previous combined datasets and 43 new overlaps. Thus, it contains a number of overlaps that is twice as large as all the previous curated datasets put together.

### Curation of sequence databases

We contributed to curate sequence databases in the following ways: 1) we added to the NCBI RefSeq genome database the proteins encoded by overlapping genes whose existence was proven but that were missing from the database. In total 10 overlaps of the dataset, i.e. one in 8, were not annotated in NCBI RefSeq (they are marked by the symbol ^§^ in S1 Table); 2) we added to the NCBI RefSeq genome database 5 overlapping genes that are experimentally proven but could not be included in the dataset because they are shorter than the cut-off of 180 nt (e.g. in Sindbis virus, Ac number NC_003215); 3) we gave access to our set of mammalian overlapping genes (S3 Table) to the curators of mammalian NCBI RefSeq [67]. Two mammalian overlaps, whose existence was proven but were not annotated in any sequence database, were added to RefSeq; 4) we corrected many sites of ribosomal frameshifting events in the NCBI reference genomes (in 7 viruses from the genus *Flavivirus* and in 19 viruses from the genus *Alphavirus*); 5) we gave access to our dataset to the curators of the database Swiss-Prot/Uniprot. They added 13 new proteins to Swiss-Prot, as well as expression or functional annotations for a number of the other overlaps, contributing to an enrichment of the ViralZone online resource (http://viralzone.expasy.org) [68].

### Overlapping genes of the dataset have a wide length distribution

**Fig 1:**
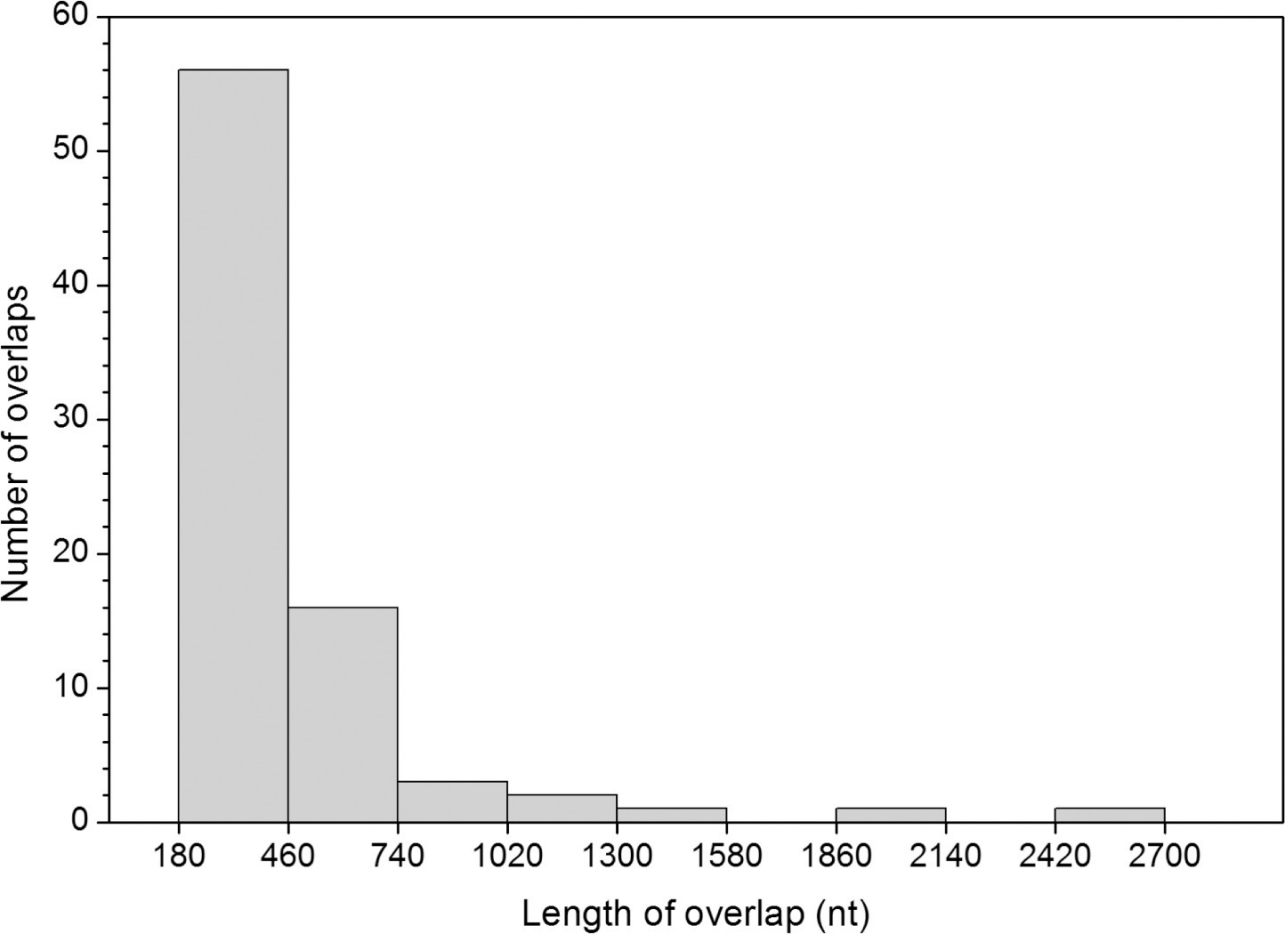
Frequency distribution of the length of the 80 overlapping genes of the dataset.

The length distribution of overlapping genes has an alpha-modal distribution (Fig 1). A large majority (60 out of 80) have a length ranging from 180 to 500 nt, corresponding to 60–166 amino acids (aa). About one fifth (15 out of 80) have a length ranging from 500 to 1000 nt. The remaining 5 overlaps have a length greater than 1000 nt, with the longest one encompassing 2,682 nt (corresponding to 894 aa) in *Alphacarmotetravirus*. The mean length of overlaps was 442 nt, with a high standard deviation (390 nt). Thus, most overlaps of our dataset encode protein regions that have a length typical of a protein domain (from 100 to 150 aa), but some encode regions that are much longer, in the length range of viral polyproteins.

### There is only a weak correlation between the length of viral genomes and that of the overlapping genes they contain

We examined the 61 viral species of the dataset to determine whether there was a relationship between the length of their genomes and of their overlapping genes. We found a weak, albeit significant, negative correlation (r = -0.27; t-Student = 2.13; P = 0.02). The trend remained, slightly increased, using the Spearman rank correlation test (rho = -34; t-Student 0 3.05; P = 0.002), confirming that it is robust. The strong correlation reported previously [42] results from having introduced a normalization and a logarithmic transformation in the calculations (see Discussion).

### A number of overlaps encode interacting proteins

A hypothesis often invoked to explain the abundance of overlapping genes in viruses is that they might endow viruses with new regulatory mechanisms, by producing viral proteins that are transcriptionally or translationally coupled, which would enable them to play complementary roles in the same pathway [26]. A particular case of proteins that take part in the same pathway is when they interact directly. We could identify 11 overlaps that encode such interacting proteins (Table 2). This number is probably an underestimate, since many proteins in the dataset have not been characterized in detail.

**Table 2:** List of the 11 pairs of overlapping genes encoding interacting proteins.

Virus species	Protein product 1	Protein product 2	Function	Bibliographic references
Adeno-associated virus 2	Capsid protein (VP1)	AAP (Assembly Activating Protein)	Virion assembly	[70]
Borna disease virus 1	X protein	Phosphoprotein	Virus replication	[77]
Chicken anemia virus	Capsid protein (VP2)	Nucleocapsid protein	Virion assembly	[69]
Chicken anemia virus	Capsid protein (VP2)	Apoptin (VP3)	Host cell apoptosis	[72, 73]
East African cassava virus	AV2 protein	Capsid protein (AV1)	Within-host virus movement ^a^	[76]
Hepatitis E virus	Phosphoprotein (ORF3)	Capsid protein (ORF2)	Virion assembly	[71]
Human papillomavirus type 16	E2 protein	E4 protein	Stabilization of the E2 protein	[78]
Influenza virus A	RNA-dependent RNA polymerase (subunit PB1)	PB1-F2 protein	Virus replication	[74]
Rotavirus A	Phosphoprotein (NSP5)	NSP6 protein	Viroplasm formation	[80, 81]
Sesbania mosaic virus	Polyprotein P2a (ATPase P10 domain)	Polyprotein P2ab (RdRp domain)	Virus replication	[75]
Simian hemorrhagic fever virus	GP3 protein	GP4 protein	Virus entry ^b^	[79]

^a^. The interaction was established in a virus species from the same genus, Cotton leaf curl Kokhran virus-Dabawali.

^b^. The interaction was established in a virus species from the same genus, Equine arteritis virus.

In 3 overlaps, the interaction between proteins encoded by overlapping genes is critical for viral assembly. They are the VP2/capsid overlap of chicken anemia virus [69], the VP1/AAP overlap of Adeno-associated 2 virus [70], and the ORF3/capsid overlap of hepatitis E virus [70]. Interestingly, the VP2 protein of chicken anemia virus also interacts with the other overlapping protein (VP3, also known as apoptin) down-regulating its apoptotic activity [72, 73].

In 2 overlaps, the interaction affects the virus replication. In influenza A virus, the F2 protein interacts with PB1, regulating its polymerase activity [74]. Likewise, in Sesbania mosaic virus, the RdRp domain of polyprotein P2ab shows an increased polymerase activity when interacting with the p10 domain of polyprotein P2a [75].

In 3 overlaps, the interaction is thought to have a role in relocating the viral genome from nucleus to cytoplasm, where assembly, egress, or movement to the neighbouring cells occurs. In Cotton leaf curl Kokhran virus-Dabawali, the interaction between AV2 and the coat protein AV1 might be implicated in cell-to-cell movement [76]. The interaction between the X protein and the phosphoprotein of Borna disease virus 1 is involved in regulating the trafficking of viral RNA from the nucleus, where replication occurs, to the cytoplasm [77]. Likewise, in human papillomavirus type 16, the interaction between E2 and E4 leads to a partial relocation of E2 from nucleus to cytoplasm [78].

In the remaining 2 cases, the interaction affects a different step of the viral cycle. GP3 and GP4 of simian hemorrhagic fever virus, together with GP2, interact to form a disulphide-linked glycoprotein complex that probably affects the viral entry in the cell [79]. In Rotavirus A, NSP5 and NSP6 interact in the viroplasm, where replication and assembly occur in the cell host [80, 81].

Proteins encoded by overlapping genes can also interact in a more indirect way, by taking part in the same biological pathway. We did not attempt an exhaustive census of these proteins but offer a few examples. In Theiler's murine encephalomyelitis virus, both the leader L protein and the L* protein inhibit the host interferon response [82, 83]. Another example is the Tax/Rex overlap in bovine leukemia virus, in which the two proteins play complementary roles [84]. Finally, a case is particularly noteworthy: in measles virus, two regions of a single gene, P, encode three proteins in overlapping frames (P, C and V). The three proteins play complementary roles (e.g. P and C or C and V), as recently summarized [28].

### The most common mechanisms to express overlapping genes occur at the level of translation

**Fig 2:**
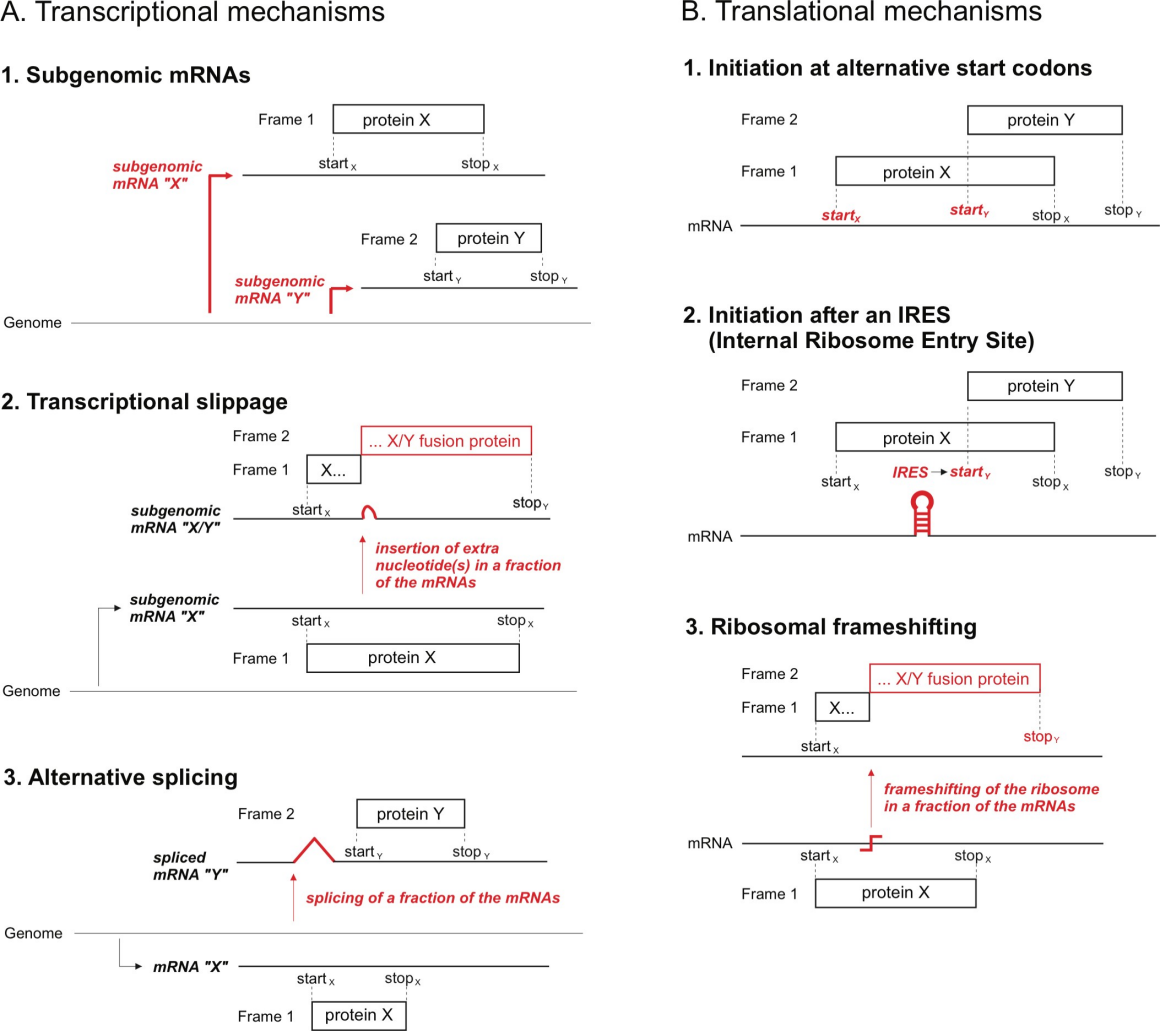
Main mechanisms used to express the proteins encoded by overlapping genes.

Overlapping genes (like all genes) express proteins by a combination of transcriptional and translational mechanisms (Fig 2). We excluded from the analysis of expression mechanisms below the 8 overlaps in which proteins are expressed by splicing (S1 Table), because 3 of them (those in which the splicing event interrupts the reading frame) were selected for their biological interest (see Methods) and, overall, this set is thus not necessarily representative of overlaps generated by splicing.

We focused on the *ultimate* mechanism that allows the expression of two proteins from the same DNA sequence. For instance, panel A1 in Fig 2 depicts a virus that produces two subgenomic RNAs, whose overlapping frames are each expressed by using their own separate start codon. In this case, we consider that the mechanism that enables their expression is the use of subgenomic RNAs, rather than the use of alternative start codons. Indeed, the alternative start codon is only accessible because of the use of a subgenomic RNA.

We could find evidence of the mechanism of expression of overlapping genes in 54 cases, and a suspected mechanism in 17 cases (Table 3). More than two thirds of overlapping genes are expressed by translational mechanisms (54 out of 71 cases, Table 3). The most common translational mechanism is the use of an alternative start codon. Other translational mechanisms include ribosomal frameshifting (7 cases) and internal ribosome entry site (IRES, 2 cases).

**Table 3:** Mechanisms of expression of overlapping genes.

Translational mechanisms (54 cases: 38 proven and 16 suspected)			Transcriptional mechanisms (17 cases: 16 proven and 1 suspected)	
Alternative start codon^a^	IRES^a^	Ribosomal frameshifting^b^	Subgenomic RNAs^a^	Transcriptional slippage^b^
29 proven and 16 suspected cases	2 proven cases	7 proven cases	12 proven and 1 suspected cases	4 proven cases

^a^Results in a completely new coding sequence

^b^Results in the fusion of a new coding sequence downstream of an existing coding sequence

The remaining third of overlapping genes is expressed by transcriptional mechanisms, among which the use of subgenomic RNAs is the most common (13 cases), followed by transcriptional slippage, also called "transcriptional editing" or “co-transcriptional nucleotide insertion” (4 cases).

From an evolutionary point of view, we note that two expression mechanisms stand out because they necessarily result in the fusion of a new coding sequence downstream of an *existing* coding sequence, rather than in the origination of a *completely new* sequence. These mechanisms are ribosomal frameshifting and transcriptional slippage. They are relatively rare, accounting for only 11 overlaps in the dataset (Table 3). Therefore, in viruses, most overlapping genes apparently result in the origination of a completely new coding sequence, rather than in a fusion of a new coding sequence to an existing one.

### Overlapping and non-overlapping genes differ significantly in their nucleotide and amino acid composition

We compared the overall composition of the overlapping coding regions with that of the non-overlapping coding regions using the chi-square test. We examined five global features, namely the composition in 1) nucleotides; 2) dinucleotides; 3) amino acids; 4) synonymous codons; and 5) amino acids with high, medium or low codon degeneracy (see Methods). The pooled set of overlapping regions (35,394 nt) differed significantly from that of non-overlapping regions (487,158 nt) for each of the five composition features examined (S4 Table). For instance, comparing the nucleotide compositions yielded a chi-square value of 745.1 (P<0.00001), which is two orders of magnitude greater than the cut-off of significance for 3 degrees of freedom (7.82; P<0.05). Comparing the other features yielded a chi-square value ranging from 360.9 (composition in amino acids with respect to the codon degeneracy; P<0.00001) to 2242.2 (composition in synonymous codons; P<0.00001).

**Fig 3:**
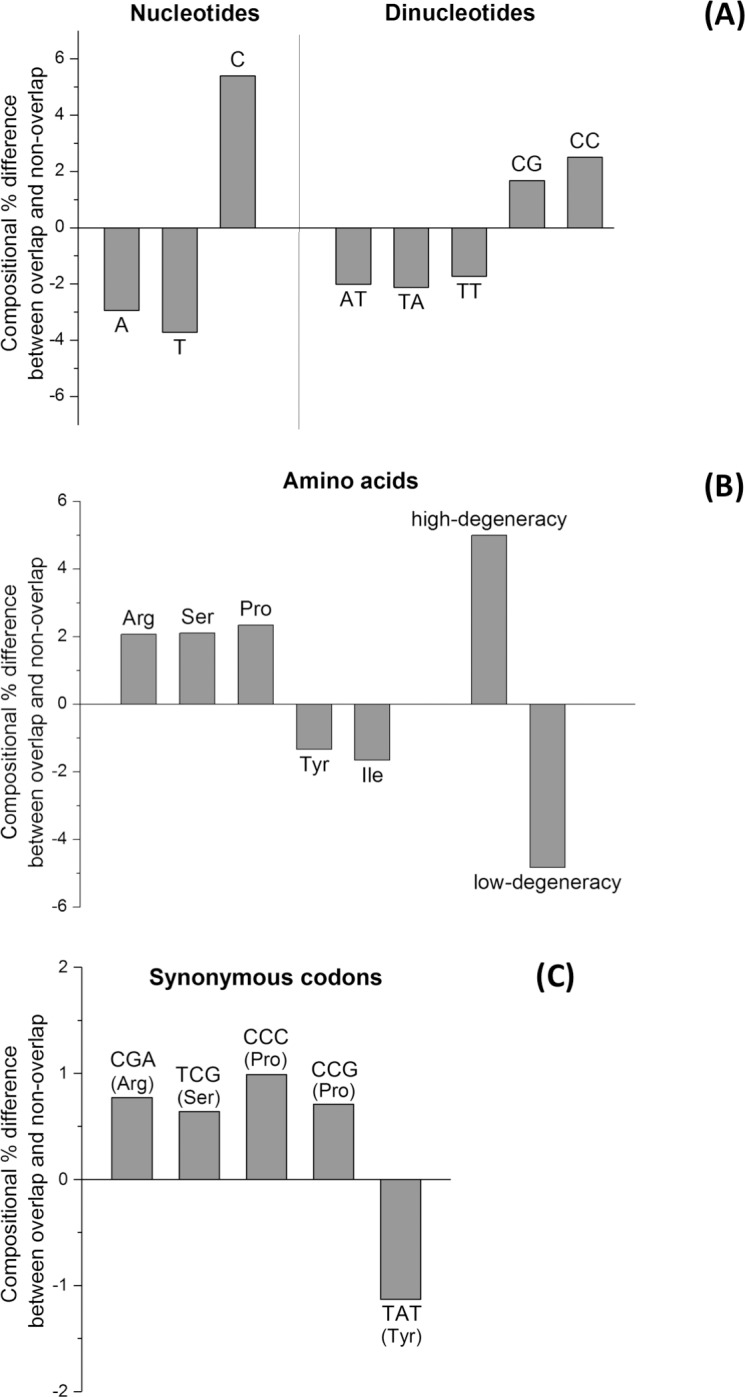
Difference between the pooled sets of overlapping and non-overlapping genes for the 20 most critical composition features. (A) Nucleotides and dinucleotides. (B) Amino acids and amino acids grouped in accordance to codon degeneracy. (C) Synonymous codons.

To identify precisely which factor contributed to the composition difference between overlapping and non-overlapping regions, we performed a more stringent chi-square test, using a cut-off value of 100.0 (P<0.00001). We identified 20 critical composition differences (Fig 3, see also S4 Table). On the one hand, overlapping genes are highly enriched in the nucleotide C, the dinucleotides CG and CC, the amino acids arginine, serine, and proline, the synonymous codons CGA, TCG, CCC, and CCG, and in amino acids with a high codon degeneracy (Fig 3). On the other hand, overlapping genes are highly depleted in the nucleotides A and T, the dinucleotides AT, TA, and TT, the amino acids tyrosine and isoleucine, the synonymous codon TAT, and in amino acids with a low codon degeneracy (Fig 3). We confirmed the relevance of the 20 critical composition differences with the Wilcoxon test for paired data. In all cases, we found a *z* score largely exceeding the cut-off of significance (z = -2.55; P <0.01) (S4 Table).

The composition biases in the 5 features we examined are clearly linked. First, regarding amino acids that are enriched, proline is encoded by codons rich in C (among which CCC and CCG, which are also enriched, see Fig 3C); likewise, arginine and serine have a high codon degeneracy, and can be encoded, respectively, by CGA and TCG, which are also enriched (Fig 3C). Second, amino acids with a low codon degeneracy, which are depleted (Fig 3B), are encoded by codons that are rich in A and T, which are depleted too (Fig 3A). Analogously, isoleucine, which is depleted (Fig 3B), is encoded by AT-rich codons (ATA, ATC, and ATT).

### Principal component analysis (PCA) of overlapping genes revealed the presence of 5 outliers

We used PCA [64–66] to evaluate whether the pattern of composition differences between the pooled sets of overlapping and non-overlapping regions was homogeneously distributed in individual overlapping genes, or if instead there were outliers with a highly atypical composition. PCA extracts the information from multiple parameters (here the 20 critical composition features we detected) and summarizes it into a much smaller set of variables (called Principal Components or PCs), with minimal loss of information.

PCA summarized the information carried by the 20 variables into three synthetic variables, that is the first (PC1), second (PC2), and third principal component (PC3). They accounted for 31.6, 29.8, and 11.7% of the total amount of variation in the source data matrix (see Methods), respectively. Taken together, the three PCs accounted for 73.1% of the total variation, i.e. the reduction from 20 to 3 variables resulted in a relatively small loss of information (26.9%).

**Fig 4:**
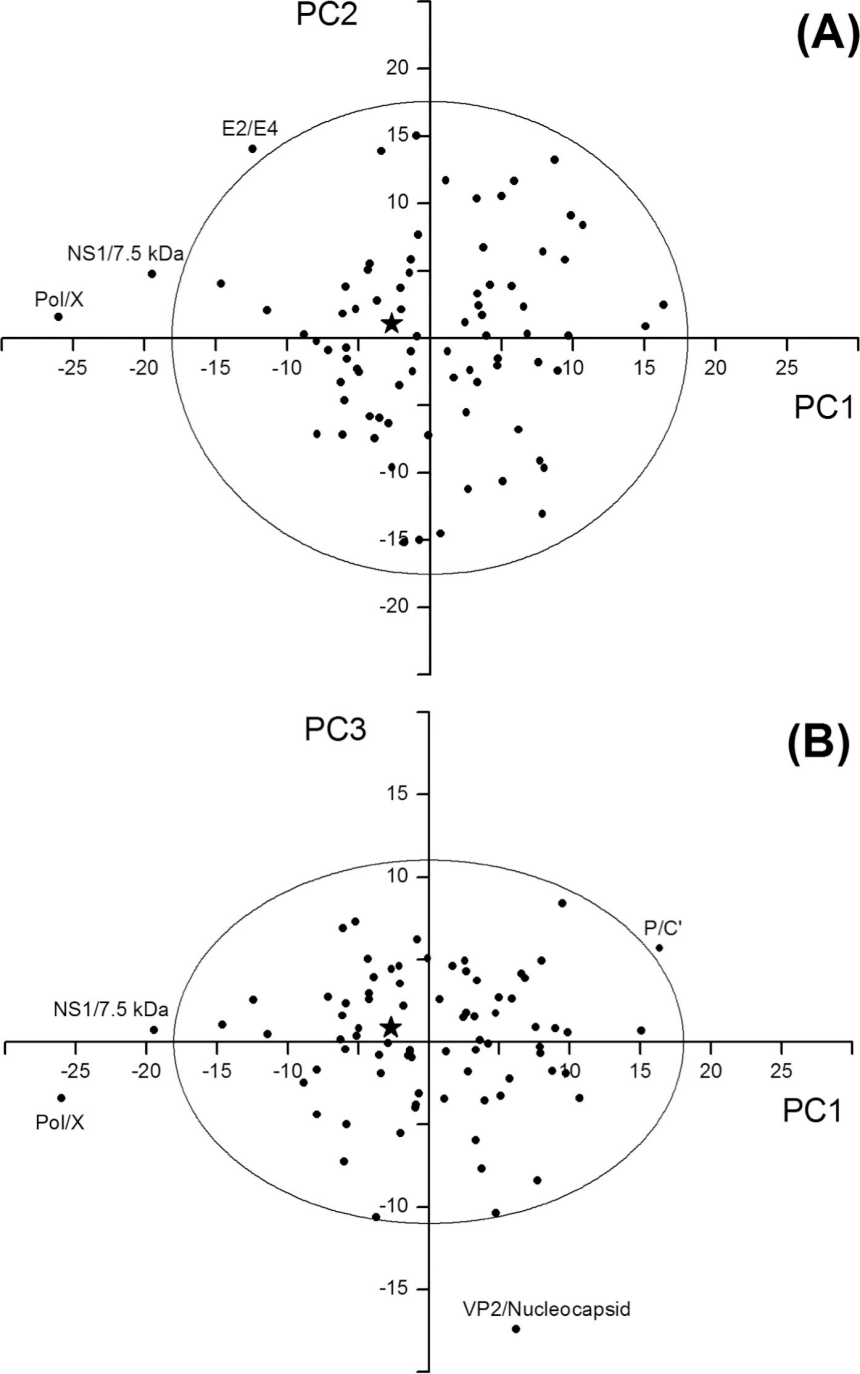
Principal component analysis (PCA) of overlapping genes. The star symbol near the origin of the axes indicates the pooled dataset of overlapping genes, while the black circles indicate the individual overlapping genes. Circles outside the ellipse are outliers, that is overlaps with a composition significantly different from the rest (P<0.05). (A) Map yielded by the first (PC1) and second (PC2) principal component. (B) Map yielded by the first (PC1) and third (PC3) principal component.

We represented the 80 overlapping genes of the dataset on two bi-dimensional maps. In the first one, PC2 was plotted against PC1 (Fig 4A), and in the second PC3 was plotted against PC1 (Fig 4B). In both maps, the star symbol located near to the origin of the axes indicates the pooled set of overlapping genes, while the black circles indicate the individual overlapping genes. Circles outside the ellipse are outliers, that is overlaps with a composition significantly different from others (P<0.05).

#### Examination of the map yielded by PC1 and PC2

The map yielded by PC1 and PC2 (Fig 4A) revealed the presence of 3 outliers. The first is the overlap Pol/X of human hepatitis B virus. It falls outside the ellipse because of a high negative PC1 score (-26.1). Three main variables contribute to PC1: the content in A, in C, and in low-degeneracy amino acids. Indeed, they have the highest (in absolute value) correlation with PC1 (r = 0.76 for A; r = -0.70 for C, and r = 0.91 for low-degeneracy amino acids, Table 4). The overlap Pol/X is thus an outlier because it has a strong depletion in A (-14.7%) and in low-degeneracy amino acids (-18.1%), and a strong enrichment in C (15.2%), with respect to the non-overlapping counterpart. This composition bias is remarkably stronger than that observed in the pooled overlapping dataset, which has a depletion in A of only -2.9%, a depletion in low-degeneracy amino acids of only -4.9%, and an enrichment in C of only 5.4% (Fig 3 and S4 Table).

**Table 4:** Correlation between the 20 critical composition features of overlapping genes and the first (PC1), second (PC2), and third (PC3) principal component.

Composition feature	PC1	PC2	PC3
**A**	0.76	0.24	-0.26
**T**	-0.18	-0.94	0.19
**C**	-0.70	0.59	0.23
**AT**	0.23	-0.70	0.09
**TA**	0.18	-0.67	-0.08
**TT**	-0.12	-0.83	0.18
**CG**	-0.41	0.41	-0.24
**CC**	-0.58	0.64	0.16
**Arginine**	0.05	0.20	-0.84
**Serine**	-0.49	-0.30	-0.03
**Proline**	-0.56	0.52	0.14
**Tyrosine**	0.18	-0.55	0.00
**Isoleucine**	0.10	-0.55	0.14
**High-degeneracy amino acids**	-0.48	-0.49	-0.60
**Low-degeneracy amino acids**	0.91	-0.02	0.16
**CGA (Arg)**	0.22	0.10	-0.61
**TCG (Ser)**	-0.18	-0.13	0.07
**CCC (Pro)**	-0.42	0.52	0.17
**CCG (Pro)**	-0.46	0.27	0.07
**TAT (Tyr)**	0.24	-0.48	-0.09

The second outlier is the overlap NS1/7.5 kDa protein of human parvovirus B19. It falls outside the ellipse because of a high negative PC1 score (-19.5). This overlap has a depletion in A (-4.8%), a strong depletion in low-degeneracy amino acids (-15.6%), and a strong enrichment in C (13.6%), compared to the non-overlapping counterpart. As in the previous case, this composition bias is remarkably stronger than that of the pooled overlapping dataset.

The third outlier was the overlap E2/E4 of human papillomavirus type 16. It falls outside the ellipse because of the joint effect of a high negative PC1 score (-12.6) and a high positive PC2 score (14.5). Three main variables contribute to PC2: the content in T, in AT, and in TT. Indeed, they have the highest (absolute value) correlation with PC2 (r = -0.94 for T; r = -0.70 for AT, and r = -0.83 for TT) (Table 4). This overlap is thus an outlier because of a strong depletion in T (-13.0%), AT (-5.8%), and TT (-5.6%), compared to the non-overlapping counterpart. Again, this composition bias is remarkably stronger than that of the pooled overlapping dataset, in which depletions in T, AT, and TT were all considerably smaller (-3.7, -2.0, and -1.7%, respectively) (Fig 3A).

#### Examination of the map yielded by PC1 and PC3

The map yielded by PC1 and PC3 (Fig 4B) revealed the presence of 2 additional outliers. The first is the overlap VP2/nucleocapsid of chicken anemia virus. It falls outside the ellipse because of a high negative PC3 score (-17.5). Only one variable, the content in arginine, mainly contributes to PC3 (r = -0.84) (Table 4). This overlap is thus an outlier because of a strong enrichment in arginine (18.6%), which is nine-fold higher than that observed in the pooled overlapping dataset (2.2%).

The other outlier is the overlap P/C’ of vesicular stomatitis Indiana virus. It falls outside the ellipse because of a joint effect of high, positive PC1 and PC3 scores. Compared to the non-overlapping counterpart, this overlap is enriched in A (3.0%) and in low-degeneracy amino acids (9.7%), and it is depleted in C (-2.3%) and in high-degeneracy amino acids (-1.0%). Unlike the 4 previous outliers, this composition bias is completely opposite to that of the pooled overlapping dataset (depletion in A and in low-degeneracy amino acids, and enrichment in C and in high-degeneracy amino acids; see Fig 3).

In summary, there are only 5 outliers out of 80 overlapping genes. 4 of them have a composition bias that goes in the same direction as that of the pooled overlapping dataset, though in a much stronger way. Only 1 outlier has a highly atypical composition bias.

### A small set of mammalian overlapping genes follows a composition bias similar to viral ones

We wanted to determine whether mammalian overlapping genes follow the same pattern of sequence composition as viral ones. Although there are few proven mammalian overlaps, we managed to identify and curate 6 of them (S3 Table).

We calculated the value of the 20 composition features in each mammalian overlap and in the non-overlapping counterpart. We used the 20 corresponding differences and the eigenvectors yielded by PCA of viral overlapping genes for obtaining the position coordinates of the 6 mammalian overlaps on PC1, PC2, and PC3. Because of the huge length of the mammalian genome with respect to that of any viral genome, we considered as non-overlapping coding region only that occurring in the mammalian gene containing the overlap.

**Fig 5:**
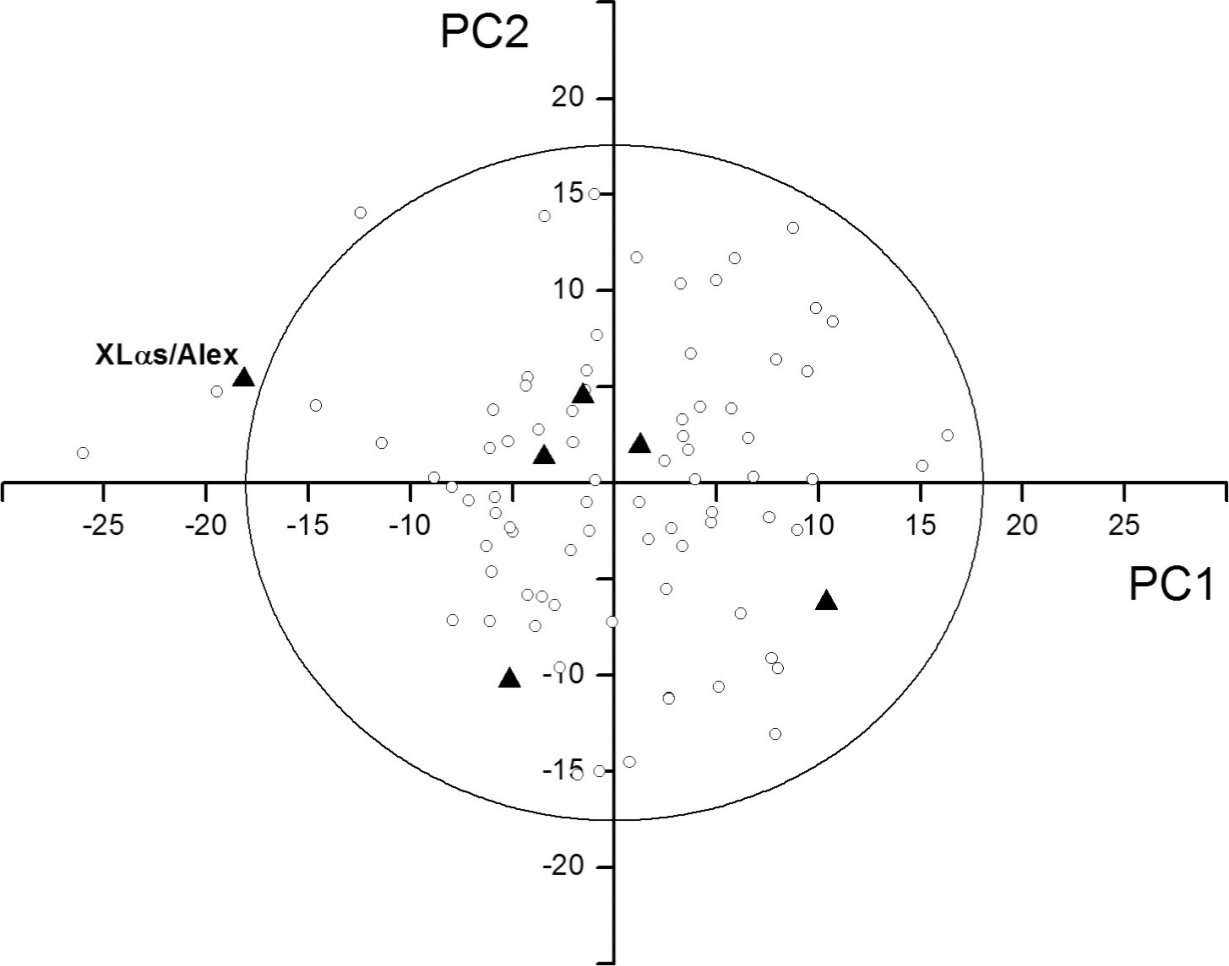
Location of the 6 mammalian overlapping genes in the PC1-PC2 map of viral overlapping genes. The mammalian overlapping genes are indicated by bold triangles, the viral overlapping genes by empty circles. The 3 circles and the triangle outside the ellipse are outliers, that is overlaps with a composition significantly different from the rest (P<0.05).

We found that 5 of the 6 mammalian overlaps fall within the ellipse in the PC1-PC2 map (bold triangles in Fig 5). The outlier is the overlap XLαs/Alex, owing to a high negative PC1 score (-18.5). Compared to the non-overlapping counterpart, it is depleted in A (-5.3%) and in low-degeneracy amino acids (-19.3%), and it is enriched in C (8.4%). Thus, this overlap shows a composition bias remarkably stronger than that of the pooled overlapping dataset, as was observed for most outliers in viruses (see above). No outlier was found in the PC1-PC3 map (data not shown).

We reported above that a number of proteins encoded by viral overlapping genes interact. Interestingly, as has been recently noted [10], some of the proteins encoded by these mammalian overlaps also interact (e.g. Alex binds XLαs [85] and ATXN1 interacts with Alt-ATXN1 [7]).

## Discussion

### Overall, overlapping genes have a peculiar composition bias, which may have a biological meaning

The first main conclusion of our study is that the overall composition of overlapping genes is significantly different from that of non-overlapping genes. Earlier studies on single virus species [25, 84, 86–88] or on single virus families [89–91] had pointed out to some composition differences. Here, by using a large and curated dataset, we reliably identified 20 composition features that are peculiar to overlapping genes (Fig 3). This pattern remained very similar, even when we used a more conservative control, which included only the non-overlapping genes closest to the overlap, instead of all non-overlapping genes (data not shown). Therefore, the composition bias of overlapping genes can be considered robust. We can distinguish two main (non-exclusive) hypotheses that could explain this composition bias.

In the first hypothesis, the composition bias would be a contributing factor (i.e. a *cause*) to the existence of overlapping genes. For instance, depletion in T, A, and TA (Fig 3A) reduces the probability of occurrence of stop codons (TGA, TAG and TAA) and thereby increases that of occurrence of long overlapping frames. An example is given by the long overlap between p69 and the replicase of Turnip yellow mosaic virus (TYMV). The viral genome has an unusually low proportion of TA dinucleotides (2.1%) including the portion of replicase that overlaps with p69 (our observations). We can thus hypothesize that the birth of the overlap was favoured by the genome composition bias of TYMV.

In the second hypothesis, the composition bias would be a *consequence* of selection acting on overlapping genes after they are born. For instance, an enrichment in amino acids with a high codon degeneracy and a depletion in amino acids with a low codon degeneracy (Fig 3B) could have been selected because they minimize the constraints under which the two overlapping reading frames evolve. This bias with respect to codon degeneracy had been suggested before [14, 92] on much smaller datasets, but not reliably proven. An example is given by the overlap between P3N-PIPO and the polyprotein of Turnip mosaic virus. Compared to the non-overlapping coding regions of the genome, the overlapping region has a comparable proportion of TA dinucleotides, which would not have resulted in a decreased probability of occurrence of stop codons, unlike in hypothesis 1. However, the overlapping region has a strong enrichment in amino acids with a high codon degeneracy (34% vs. 21%), which might be a sign of selection having acted to minimize the constraints under which the two overlapping frames evolve (hypothesis 2).

Another composition bias that may minimize structural constraints in proteins encoded by overlapping genes is an enrichment in amino acids favouring structural disorder [14]. In agreement with this proposal, arginine, serine and proline, which are highly enriched in proteins encoded by overlapping genes (Fig 3B), all promote structural disorder [93].

Of course, we cannot exclude a "mixed" scenario, in which the overlapping region had a composition different from the rest of the genome (i.e. rich in amino acids with a high codon degeneracy) *before* the birth of the overlap. This "preoptimized" composition might have favoured the retention of the overlap *after* its birth, by minimizing the selection constraints acting on it.

### The vast majority of overlapping genes follow the same pattern of sequence composition

The second main conclusion of our study is that the vast majority of overlapping genes (75 out of 80) follow a common pattern of sequence composition (Fig 4). We found only 5 outliers, 4 of which have a composition bias going in the same direction as that of the pooled overlapping dataset, though in a much stronger way (Fig 4). This pattern was confirmed even when we used as control the non-overlapping genes closest to the overlap, instead of all non-overlapping genes (data not shown).

We also found that 5 out of 6 mammalian overlapping genes follow the same pattern of sequence composition as viral ones, and that the only mammalian outlier has a composition bias similar to that of viral outliers (Fig 5). Thus, mammalian overlaps may have composition features similar to that of viral ones, though this finding must be confirmed on a larger dataset.

### The length of viral genomes and of their overlapping genes are only weakly correlated, in genomes up to 30 kb

Chirico et al. [42] previously reported a strong, negative correlation between the length of viral genomes and that of the overlapping genes they contain: to estimate a figure comparable with our dataset, we pooled their data concerning non-bacterial RNA and DNA virus with genomic length below 30 kb and we obtained r = -0.56 (not shown). In contrast, we find a weak, albeit significant, negative correlation (r = -0.27), confirmed when using Spearman correlation coefficient (rho = -0.34). The discrepancy is due to the use of a normalization and of a logarithmic transformation in the calculations of Chirico and co-workers [42]. Indeed, they calculate the correlation between the length of the genome and the *ratio* of the length of overlaps to the length of the genome. Considering the same variable (the genome length) twice in a correlation test will necessary produce an artefactual correlation, since the data examined are not independent. In fact, we note that by using the untransformed pooled genomic subset (that is excluding bacteriophages and genomes >30 kb) from Chirico et al. [42], without normalization, a correlation coefficient of r = -0.27 is obtained (not shown), which is identical to the one reported here.

Our results are only valid for genomes smaller than 30 kb (the upper threshold for inclusion of our dataset). Yet Brandes & Linial [43] recently reported the same weak correlation as we did, on a dataset containing all known viral genomes, up to genomes of one million bases in size. However, we think that such large viral genomes might contain overlooked overlapping genes, for at least three reasons: 1) in large viruses, each individual gene is usually the subject of much less attention than in small or medium-sized viruses, as large viruses contain dozens, or even hundreds of times more genes; 2) overlapping genes shorter than a certain threshold (e.g. 300 nt) are often not annotated in the genome sequences; 3) we could find no publication that reports the use of computational tools, such as Synplot2 [53], to detect overlapping genes in the genome of large viruses, unlike that of small viruses.

Accordingly, recent experimental studies have revealed numerous putative overlapping genes in large viruses such as herpesvirus [94] and poxvirus [95]. Therefore, we think that the weak, negative correlation between the length of viral genomes and the length of their overlapping genes, which we and Brandes & Linial [43] report, can only be considered proven for genomes shorter than 30 kb.

### Limitations of our study

A first limitation of our study is that we focused only on overlapping genes from small or medium-sized eukaryotic viruses, because we were aware that overlapping genes were not reliably detected in the genomes of large eukaryotic viruses. In retrospect, our decision is justified by the discovery mentioned above, occurred during our study, of numerous putative overlapping genes in herpesvirus [94] and poxvirus [95], thanks to recent advances in genome-wide ribosome profiling techniques [96].

A second limitation is that our results might not be applicable to antiparallel overlapping genes. We are aware only of 3 proven such genes that fit the size criteria of our dataset. These might be more frequent than currently thought, as attested by recent sequence analyses that identified an antisense coding sequence, termed ASP, under clear selection in human immunodeficiency virus type 1 [97].

### Implications of our study

Our study has a number of implications for the community. First, our dataset is a useful starting point for much-needed systematic studies on overlapping genes. For instance, since the vast majority of overlaps have at least one homolog (data not shown), we could investigate their evolution through comparative analyses of homologous overlapping sequences. Second, we have identified 6 overlaps with an unusual sequence composition (see outliers in Figs 4 and 5). Further studies need to identify whether this composition is linked to their function. Third, 8 viral overlapping genes, for which there is incomplete evidence need to be confirmed experimentally (S2 Table).

Fourth, the composition bias discovered here might prove useful to detect overlapping genes, since we now have in hand 20 variables whose ability to discriminate between dual- and single-coding regions can be systematically assessed. Having found that PCA has a poor ability to separate overlapping genes from non-overlapping genes (S1 Fig), we believe that potential methods to exploit these variables include the multivariate statistical methods that maximize the variance between groups and minimize the variance within groups (e.g. the linear and quadratic discriminant functions).

Finally, this study confirms that a change in practice of viral genome annotation is necessary, as overlapping genes are present in most viral families that we sampled. Researchers who sequence viral genomes need to pay closer attention to overlapping genes. This means systematically detecting, by computational methods such as Synplot2 [53], overlapping genes that are likely to be expressed. In addition, researchers need to deposit experimentally proven overlaps in databases, which is not the case at present: in our study, several proven overlaps were not annotated in reference databases, or even in any database.

## Supporting information

S1 Fig Principal component analysis (PCA) of overlapping and non-overlapping genes. We carried out PCA on a matrix of 160 rows (the 80 overlapping genes of our dataset and the 80 corresponding non-overlapping genes in the virus genome) and 20 columns (the 20 critical composition features). Black circles indicate the 80 overlapping genes and red circles the 80 non-overlapping genes. PC1, PC2, and PC3 account for 54.8 18.1, and 9.7% of the total amount of variation in the source data matrix, respectively. (A) Map yielded by the first (PC1) and second (PC2) principal component. (B) Map yielded by the first (PC1) and third (PC3) principal component.
(TIF)

S1 Table List of the 80 viral proven overlapping genes assembled in S1 Dataset. The genes are grouped in 7 tables (from S1a to S1g) in accordance to the nature of the virus genome.
(DOC)

S2 Table List of the 8 viral overlapping genes for which there is only partial experimental evidence. (DOC)

S3 Table List of the 6 experimentally proven mammalian overlapping genes assembled and analysed in this work. (DOC)

S4 Table Details of the comparative analysis of overlapping and non-overlapping genes. S4a Table shows the comparison of the pooled dataset of overlapping regions with that of the non-overlapping regions for 5 composition features. S4b Table lists the the 20 critical composition features peculiar to the overlapping gene dataset (chi-square > 100.0; 1 degree of freedom; P<0.00001; *z* score < -2.55; P <0.01).
(DOC)

S1 Dataset Dataset of 80 proven overlapping genes from eukaryotic viruses. (XLS)
