# Supplementary figures and images for: Overlapping genes and the proteins they encode differ significantly in their sequence composition from non-overlapping genes
Source: PLoS One. 2018 Oct 19;13(10):e0202513. doi: 10.1371/journal.pone.0202513 (PMC6195259; doi:10.1371/journal.pone.0202513)

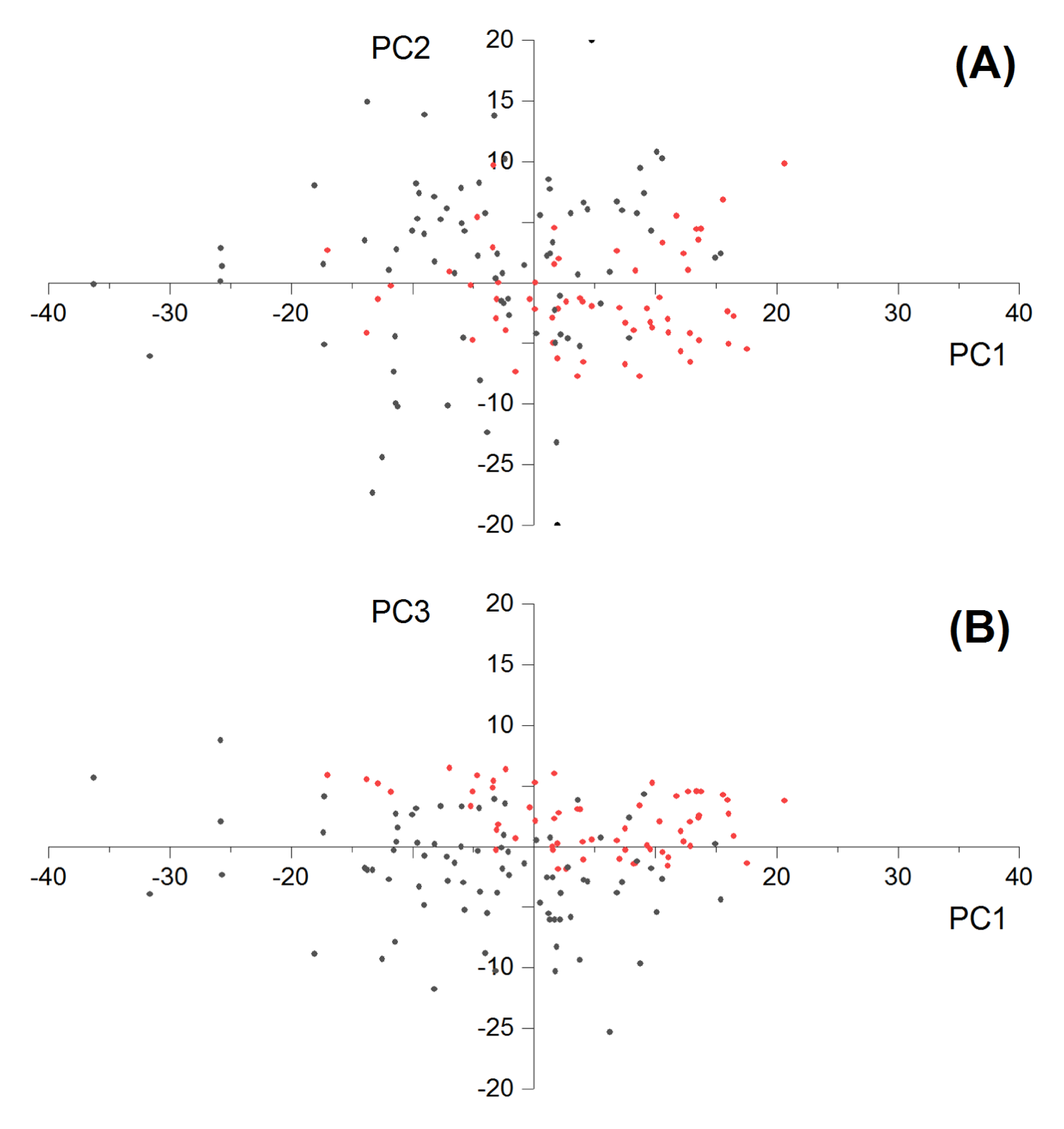

Supplement: S1 Fig — We carried out PCA on a matrix of 160 rows (the 80 overlapping genes of our dataset and the 80 corresponding non-overlapping genes in the virus genome) and 20 columns (the 20 critical composition features). Black circles indicate the 80 overlapping genes and red circles the 80 non-overlapping genes. PC1, PC2, and PC3 account for 54.8 18.1, and 9.7% of the total amount of variation in the source data matrix, respectively. (A) Map yielded by the first (PC1) and second (PC2) principal component. (B) Map yielded by the first (PC1) and third (PC3) principal component. (TIF) [file pone.0202513.s001.tif]
