# Supplementary material for: Overlapping genes and the proteins they encode differ significantly in their sequence composition from non-overlapping genes
Source: PLoS One. 2018 Oct 19;13(10):e0202513. doi: 10.1371/journal.pone.0202513 (PMC6195259; doi:10.1371/journal.pone.0202513)
Supplement: S1 Table — The genes are grouped in 7 tables (from S1a to S1g) in accordance to the nature of the virus genome. (DOC) [file pone.0202513.s002.doc]

**S1 Table. List of the 80 viral proven overlapping genes assembled in S1 Dataset.** The genes are grouped in 7 tables (from S1a to S1g) in accordance to the nature of the virus genome.

**S1a Table.** **37 pairs of overlapping genes from 26 ssRNA+ viruses.** The (^§^) symbol in the column “Genome Ac number” indicates the overlapping genes that were added by the authors to the NCBI reference genome database.

| Family  (-viridae) | Genus  (-virus) | Virus species | Genome  Ac number | Protein products | Protein  Ac numbers |
| --- | --- | --- | --- | --- | --- |
| Arteri | Simarteri | Simian hemorrhagic fever virus | NC_003092 | nsp2/nsp2TF | YP_009109556/YP_009172490 |
| Arteri | Simarteri | Simian hemorrhagic fever virus | NC_003092 | E/GP2 | YP_009037600/NP_203547 |
| Arteri | Simarteri | Simian hemorrhagic fever virus | NC_003092 | GP3/GP4 | NP_203548/NP_203549 |
| Arteri | Simarteri | Simian hemorrhagic fever virus | NC_003092 | GP5/5a | NP_203550/YP_009037601 |
| Betaflexi | Tricho | Apple chlorotic leaf spot virus | NC_001409 | movement protein/ capsid protein | NP_040552/NP_040553 |
| Betaflexi | Capillo | Apple stem grooving virus | NC_001749 | polyprotein/movement protein (36kDa protein) | NP_044335/NP_044336 |
| Bromo | Ilar | Spinach latent virus | NC_003809 | RNA-dependent RNA polymerase (2a) /2b | NP_620678/NP_620679 |
| Bromo | Cucumo | Cucumber mosaic virus | NC_002035 | RNA-dependent RNA polymerase (2a)/ 2b | NP_049324/NP_619631 |
| Calici | Noro | Murine norovirus | NC_008311 (^§^) | capsid protein (VP1)/ VF1 (virulence factor 1) | YP_720002/YP_006390081 |
| Carmotetra | Alphacarmo-tetra | Providence virus | NC_014126 | p130/replicase (p104) | YP_003620396/YP_003620397 |
| Corona | Betacorona | SARS coronavirus | NC_004718 | 3a/3b | NP_828852/NP_828853 |
| Corona | Betacorona | SARS coronavirus | NC_004718 | nucleocapsid protein/ 9b | NP_828858/NP_828859 |
| Dicistro | Apara | Israel acute paralysis virus | NC_009025 (^§^) | capsid protein/ORFx (Pog) | YP_001040003/YP_006390080 |
| Flavi | Hepaci | Hepatitis C virus | NC_004102 | polyprotein/F (ARFP) | NP_671491/NP_803170 |
| Hepe | Orthohepe | Hepatitis E virus | NC_001434 | phosphoprotein (ORF3)/ capsid protein (ORF2) | YP_003864075/NP_056788 |
| Luteo | Polero | Potato leafroll virus | NC_001747 | P0/RNA-dependent RNA polymerase | NP_056746/NP_056748 |
| Luteo | Polero | Potato leafroll virus | NC_001747 | P1/RNA-dependent RNA polymerase | NP_056747/NP_056748 |
| Luteo | Polero | Potato leafroll virus | NC_001747 | capsid protein (P3)/ movement protein (P4) | NP_056749/NP_056750 |
| Luteo | Enamo | Pea enation mosaic virus | NC_003629 | P0/RNA-dependent RNA polymerase | NP_619735/NP_620026 |
| Noda | Alphanoda | Flock house virus | NC_004146 | RNA-dependent RNA polymerase (A) /B2 | NP_689444/NP_689446 |
| Noda | Betanoda | Striped Jack nervous necrosis virus | NC_003448 | RNA-dependent RNA polymerase (A)/B2 (B) | NP_599247/NP_599248 |
| Picorna | Cardio | Encephalomyo-carditis virus | NC_001479 (^§^) | polyprotein/2B* | NP_056777/YP_006383903 |
| Picorna | Cardio | Theiler's murine encephalo-myelitis virus | NC_001366 | polyprotein/L* | NP_040350/YP_003587920 |
| Poty | Poty | Sweet potato feathery mottle virus | NC_001841 (^§^) | polyprotein/P1N-PISPO | NP_045216/YP_009440977 |
| Poty | Poty | Turnip mosaic virus | NC_002509 | polyprotein/P3N-PIPO | NP_062866/YP_003587806 |
| Tombus | Betacarmo | Hibiscus chlorotic ringspot virus | NC_003608 | p28/p23 | NP_619672/NP_619673 |
| Tombus | Betacarmo | Hibiscus chlorotic ringspot virus | NC_003608 | capsid protein/p25 | NP_619676/NP_619677 |
| Tombus | Machlomo | Maize chlorotic mottle virus | NC_003627 | p32/p50 | NP_619717/NP_619719 |
| Tombus | Machlomo | Maize chlorotic mottle virus | NC_003627 | p31/p7b | NP_619720/YP_009237216 |
| Tombus | Machlomo | Maize chlorotic mottle virus | NC_003627 | p31/capsid protein | NP_619720/NP_619722 |
| Tombus | Panico | Panicum mosaic virus | NC_002598 | capsid protein (p26)/p15 | NP_068346/NP_068347 |
| Tombus | Tombus | Tomato bushy stunt virus | NC_001554 | p22/p19 | NP_062900/NP_062901 |
| Tombus | Umbra | Tobacco bushy top virus | NC_004366 | movement protein (ORF3)/movement protein (ORF4) | NP_733849/NP_733850 |
| Tymo | Tymo | Turnip yellow mosaic virus | NC_004063 | movement protein (p69)/replicase | NP_663296/NP_663297 |
| Unassigned | Sobemo | Sesbania mosaic virus | NC_002568 | Px/polyprotein P2ab (protease domain) | YP_008873690/NP_066393 |
| Unassigned | Sobemo | Sesbania mosaic virus | NC_002568 | polyprotein P2a (ATPase P10 domain)/polyprotein P2ab (RdRp domain) | NP_066392/NP_066393 |
| Unassigned | Sobemo | Sesbania mosaic virus | NC_002568 | polyprotein P2ab (RdRp domain)/capsid protein | NP_066393/NP_066394 |

**S1b Table.** **15 pairs of overlapping genes from 13 ssRNA- viruses.** The (^§^) symbol in the column “Genome Ac number” indicates the overlapping genes that were added by the authors to the NCBI reference genome database.

| Family  (-viridae) | Genus  (-virus) | Virus species | Genome  Ac number | Protein products | Protein  Ac numbers |
| --- | --- | --- | --- | --- | --- |
| Borna | Borna | Borna disease virus 1 | NC_001607 | X protein/ phosphoprotein (P) | YP_009272535/NP_042021 |
| Filo | Ebola | Zaire ebolavirus | NC_002549 | secreted glycoprotein (sGP)/ envelope glycoprotein (GP1,2) | NP_066247/NP_066246 |
| Hantaviridae | Orthohanta | Puumala virus | NC_005224 | nucleocapsid protein/non- structural protein NSs | NP_941984/YP_004928150 |
| Orthomyxo | InfluenzaA | Influenza A virus | NC_002021 | RNA-dependent RNA polymerase (subunit PB1)/PB1-F2 | NP_040985/YP_418248 |
| Orthomyxo | InfluenzaA | Influenza A virus | NC_002022 (^§^) | RNA-dependent RNA polymerase (subunit PA)/PA-X | NP_040986/YP_006495785 |
| Orthomyxo | InfluenzaB | Influenza B virus | NC_002209 | glycoprotein NB/neuraminidase | NP_056662/NP_056663 |
| Orthomyxo | Isa | Infectious salmon anemia virus | NC_006497 | P6 (ORF2)/P7 (ORF1) | YP_145796/YP_145797 |
| Paramyxo | Morbilli | Measles virus | NC_001498 | phosphoprotein (P)/C | NP_056919/NP_056920 |
| Paramyxo | Morbilli | Measles virus | NC_001498 (^§^) | phosphoprotein (P)/V | NP_056919/YP_003873249 |
| Paramyxo | Respiro | Sendai virus | NC_001552 | C’/phosphoprotein (P) | NP_056872/NP_056873 |
| Peribunya | Orthobunya | La Crosse virus | NC_004110 | nucleocapsid protein/non-structural protein NSs | NP_671970/NP_671971 |
| Pneumo | Orthopneu-mo | Pneumonia virus of mice J3666 | NC_006579 | phosphoprotein (P)/ P2 | YP_173327/YP_173328 |
| Rhabdo | Cytorhabdo | Barley yellow striate mosaic virus | NC_028244 | ORF4 protein/ORF5 protein | YP_009177225/YP_009177226 |
| Rhabdo | Vesiculo | Vesicular stomatitis Indiana virus | NC_001560 | phosphoprotein (P)/C’ | NP_041713/YP_003587923 |
| Rhabdo | Vesiculo | Vesicular stomatitis New Jersey virus | NC_024473 (^§^) | phosphoprotein (P)/C’ | YP_009047082/YP_009440976 |

**S1c Table.** **14 pairs of overlapping genes from 9 ssDNA viruses.** The (^§^) symbol in the column “Genome Ac number” indicate the overlapping genes that were added by the authors to the NCBI reference genome database. The (**) symbol in the column “Genome Ac number” indicates overlapping genes generated by alternative splicing but without interruption of the reading frame.

| Family  (-viridae) | Genus  (-virus) | Virus species | Genome  Ac number | Protein products | Protein  Ac numbers |
| --- | --- | --- | --- | --- | --- |
| Anello | Gyro | Chicken anemia virus | NC_001427 | capsid protein (VP2)/apoptin (VP3) | NP_056773/NP_056774 |
| Anello | Gyro | Chicken anemia virus | NC_001427 | capsid protein (VP2)/nucleocapsid protein | NP_056773/NP_056775 |
| Gemini | Begomo | East African cassava mosaic virus | NC_004674 | AV2 protein/capsid protein (AV1) | NP_817107/NP_817108 |
| Gemini | Begomo | East African cassava mosaic virus | NC_004674 | transcriptional activator (TrAP, AC2)/replication enhancer (Ren, AC3) | NP_817111/NP_817110 |
| Gemini | Begomo | East African cassava mosaic virus | NC_004674 | replication associated protein (Rep, AC1)/AC4 | NP_817112/NP_817113 |
| Gemini | Curto | Beet curly top virus | NC_001412 | movement protein (V3)/V2 | NP_899663/NP_040558 |
| Gemini | Curto | Beet curly top virus | NC_001412 | C1/C2 | NP_040557/NP_040561 |
| Parvo | Bocaparvo | Canine minute virus | NC_004442 (**) | NS1/NP1 | NP_758521/NP_758522 |
| Parvo | Brevidenso | Aedes albopictus densovirus | NC_004285 | NS1/NS2 | NP_694827/NP_694828 |
| Parvo | Dependoparvo | Adeno-associated virus-2 | NC_001401 | capsid protein (VP1)/AAP (Assembly Activating Protein) | YP_680426/YP_004030758 |
| Parvo | Dependoparvo | Adeno-associated virus-2 | NC_001401 | capsid protein (VP1)/X protein | YP_680426/YP_009110690 |
| Parvo | Erythroparvo | Human parvovirus B19 | NC_000883 (**) | NS1/7.5 kDa protein | YP_004928144/YP_004928145 |
| Parvo | Protoparvo | Porcine parvovirus | NC_001718 (^§^) | capsid protein (VP2)/SAT | NP_757372/YP_006355433 |
| Parvo | Iteradenso | Dendrolimus punctatus densovirus | NC_006555 | NS1/NS2 | YP_164339/YP_164340 |

**S1d Table.** **5 pairs of overlapping genes from 5 dsRNA viruses.** The (^§^) symbol in the column “Genome Ac number” indicates the overlapping genes that were added by the authors to the NCBI reference genome database.

| Family  (-viridae) | Genus  (-virus) | Virus species | Genome  Ac number | Protein products | Protein  Ac numbers |
| --- | --- | --- | --- | --- | --- |
| Birna | Aquabirna | Infectious pancreatic necrosis virus | NC_001915 | VP5/polyprotein | NP_047195/NP_047196 |
| Reo | Orthoreo | Mammalian orthoreovirus | NC_013231 (^§^) | sigma1 (outer capsid protein,hemagglutinin)/sigma1s (minor capsid cell-attachment protein) | NP_694682/YP_009344826 |
| Reo | Orbi | Bluetongue virus | NC_006008 (^§^) | VP6/NS4 | YP_052953/YP_006390083 |
| Reo | Rota | Rotavirus A | NC_011505 | phosphoprotein (NSP5)/NSP6 | YP_002302224/YP_002302225 |
| Reo | Phyto | Rice dwarf virus | NC_003768 | Pns12/Pns12-OP | NP_620538/YP_003587924 |

**S1e Table.** **1 pair of overlapping genes from a dsDNA virus.** The double asterisk in the column “Genome Ac number” indicates overlapping genes generated by alternative splicing but without frame interruption.

| Family  (-viridae) | Genus  (-virus) | Virus species | Genome  Ac number | Protein products | Protein  Ac numbers |
| --- | --- | --- | --- | --- | --- |
| Papilloma | Alphapapilloma | Human papillomavirus type 16 | NC_001526 (**) | E2/E4 | NP_041328/YP_009268708 |

**S1f Table.** **6 pairs of overlapping genes from 6 ssRNA-RT viruses.** The single asterisk in the column “Genome Ac number” indicates overlapping genes in which at least one frame is interrupted by splicing. The (**) symbol in the column “Genome Ac number” indicates overlapping genes generated by alternative splicing but without interruption of the reading frame.

| Family  (-viridae) | Genus  (-virus) | Virus species | Genome  Ac number | Protein products | Protein  Ac numbers |
| --- | --- | --- | --- | --- | --- |
| Retro | Deltaretro | Bovine leukemia virus | NC_001414 (*) | rex protein/tax protein | NC_056898/NC_056900 |
| Retro | Lenti | Human immunodeficiency virus type 1 | NC_001802 | gag protein (p6 domain)/ pol protein (p6* domain) | NP_057850/NP_057849 |
| Retro | Lenti | Human immunodeficiency virus type 2 | KU179861 (*) | env protein/rev protein | ALQ56962/ALQ56964 |
| Retro | Lenti | Simian immunodeficiency virus | NC_001549 (**) | vif protein/vpx protein | NP_054370/NP_054371 |
| Retro | Lenti | Simian immunodeficiency virus SIV-mnd 2 | NC_004455 (**) | env protein/nef protein | NP_758892/NP_758893 |
| Retro | Spuma | Feline foamy virus | NC_001871 (*) | bel protein/bet protein | NP_056917/NP_056916 |

**S1g Table.** **2 pairs of overlapping genes from a dsDNA-RT virus**

| Family  (-viridae) | Genus  (-virus) | Virus species | Genome  Ac number | Protein products | Protein  Ac numbers |
| --- | --- | --- | --- | --- | --- |
| Hepadna | Orthohepadna | Hepatitis B | NC_003977 | polymerase (P) /X protein | YP_009173866/YP_009173867 |
| Hepadna | Orthohepadna | Hepatitis B | NC_003977 | polymerase (P) /large envelope protein (L) | YP_009173866/YP_009173869 |
