# Supplementary material for: Overlapping genes and the proteins they encode differ significantly in their sequence composition from non-overlapping genes
Source: PLoS One. 2018 Oct 19;13(10):e0202513. doi: 10.1371/journal.pone.0202513 (PMC6195259; doi:10.1371/journal.pone.0202513)
Supplement: S2 Table — (DOC) [file pone.0202513.s003.doc]

**S2 Table. List of the 8 viral** overlapping genes for which there is only partial experimental evidence

| **Family**  **(-viridae)** | **Genus**  **(-virus)** | **Virus species** | **Genome**  **Ac number** | **Protein products** | **Protein**  **Ac numbers** | **Boundaries of overlapping genes ^a^** |
| --- | --- | --- | --- | --- | --- | --- |
| Alphaflexi | Mandari | Indian citrus ringspot virus | NC_003093 | capsid protein/putative 23 kDa nucleic acid binding protein | NC_203557/NC_203558 | 6854-7156 6856-7158 |
| Alphatetra | Omegatetra | Dendrolimus punctatus tetravirus | NC_005899 | p17/capsid protein p71 | YP_025095/YP_025096 | 372-755 374-757 |
| Arteri | Simarteri | Simian hemorrhagic fever virus | NC_003092 | GP2’/E’ | NP_203544/YP_009037599 | 11046-11333 11047-11331 |
| Arteri | Simarteri | Simian hemorrhagic fever virus | NC_003092 | GP2’/GP3’ | NP_203544/NP_203545 | 11484-11798 11486-11800 |
| Barna | Barna | Mushroom bacilliform virus | NC_001633 | hypothetical protein (ORF1)/hypothetical protein (ORF2) | NP_042508/NP_042509 | 67-600 68-601 |
| Barna | Barna | Mushroom bacilliform virus | NC_001633 | hypothetical protein (ORF2)/putative RNA-dependent RNA polymerase (ORF3) | NP_042509/NP_042510 | 1592-2041 1594-2043 |
| Flavi | Flavi | Culex flavivirus | NC_008604 | polyprotein (NS2A-NS2B)/truncated polyprotein (NS2AN-FIFO) | YP_899469/YP_006470608 | 3410-4294 3412-4293 |
| Reo | Oryza | Rice ragged stunt virus | NC_003771 | RNA-dependent RNA polymerase/36.9 kDa protein (P4b) | NP_620541/NP_620542 | 489-1472 491-1471 |

^a^. The boundaries are given as the nucleotide position in the viral genome.
