# Supplementary material for: Overlapping genes and the proteins they encode differ significantly in their sequence composition from non-overlapping genes
Source: PLoS One. 2018 Oct 19;13(10):e0202513. doi: 10.1371/journal.pone.0202513 (PMC6195259; doi:10.1371/journal.pone.0202513)
Supplement: S3 Table — (DOC) [file pone.0202513.s004.doc]

**S3 Table. List of the 6 experimentally proven mammalian overlapping genes assembled and analysed in this work.**

| Gene | mRNA RefSeq ac number (and splice variant ac number if applicable) | Mechanism of expression (^§^) | Length of mRNA (nt) | Overlapping genes | Length of overlap (nt) | Boundaries of overlapping coding regions (on mRNA) | Boundaries of non-overlapping coding regions (on mRNA) | References for existence of the overlap and its boundaries |
| --- | --- | --- | --- | --- | --- | --- | --- | --- |
| LBHD1 - LBH domain containing 1 (human) | NM_024099  mRNA expressing LBDH1 | Overlap generated by alternative splicing | 1357 | LBHD1/C11orf98 | 255 |  | *217-753: LBDH1 | [1-3] |
|  | NM_001286086  Variant 1 expressing C11orf98 |  | 666 |  |  | *86-340: LBDH1  87-341: C11orf98 | *48-86: C11orf98 |  |
| ATXN1 - ataxin 1 (human) | NM_000332  Variant 1 representing ATXN1  NM_001357857  Variant 1 representing Alt-ATXN1 | Overlap generated by alternative start codon | 10636 | ATXN1/Alt-ATXN1 | 561 | 999-1559: ATXN1 1001-1558: Alt-ATXN1 | 972-998: ATXN1 1560-3419: ATXN1 | [4] |
| PRNP - prion protein (human) | NM_183079  Variant 2 representing PrP  NM_001271561  Variant 2 representing Alt-PrP | Overlap generated by alternative start codon | 2804 | PrP/Alt-PrP | 225 | 513-737: PrP  515-736: Alt-PrP | 426-512: PrP  738-1187: PrP | [5] |
| GNAS - GNAS complex locus (human) | NM_080425  Variant 2 representing XLαs  NM_001077490  Variant 2 representing Alex | Overlap generated by alternative start codon | 3784 | XLαs /Alex | 1884 | 472-2355: XLαs  473-2353: Alex | 286-471: XLαs 2356-3399: XLαs | [6, 7] |
| Adora2a - adenosine A2a receptor (mouse) | NM_001357942  mRNA representing uORF5  NM_053294  mRNA representing Adora2a | Overlap generated by alternative start codon | 2491 | uORF5/Adora2a | 342 | 427-768: uORF5  429-770: Adora2a | 364-426: uORF5 771-1661: Adora2a | [8] |
| Cdkn2a - cyclic dependent kinase inhibitor 2A (mouse) | NM_001040654  Variant 2 expressing p16INK4a | Overlap generated by alternative splicing | 850 | P16INK4a/P19ARF | 198 | *205-528: P16INK4a 207-527: P19ARF | *82-204: P16INK4a 529-588: P16INK4a | [9] |
|  | NM_009877  Variant 1 expressing p19ARF |  | 929 |  |  |  | *97-285:  P19ARF |  |

(^§^) We list here the ultimate mechanism that results in the expression of two proteins from the same DNA sequence (see Results, paragraph on mechanism expression).

* Since the overlap is generated by an alternative splicing event, the transcript expressing one of the two frames does not contain enough non-overlapping coding region to reliably compare the compositional features; thus, some or all of boundaries of the non-overlapping regions of the latter refer to another transcript expressing it, as indicated in the table.

**References**

1. Oyama M, Kozuka-Hata H, Suzuki Y, Semba K, Yamamoto T, Sugano S. Diversity of translation start sites may define increased complexity of the human short ORFeome. Mol Cell Proteomics. 2007;6(6):1000-6. doi: 10.1074/mcp.M600297-MCP200.

2. Michel AM, Choudhury KR, Firth AE, Ingolia NT, Atkins JF, Baranov PV. Observation of dually decoded regions of the human genome using ribosome profiling data. Genome Res. 2012;22(11):2219-29. doi: 10.1101/gr.133249.111.

3. Chu Q, Rathore A, Diedrich JK, Donaldson CJ, Yates JR, 3rd, Saghatelian A. Identification of Microprotein-Protein Interactions via APEX Tagging. Biochem. 2017;56(26):3299-306. doi: 10.1021/acs.biochem.7b00265.

4. Bergeron D, Lapointe C, Bissonnette C, Tremblay G, Motard J, Roucou X. An out-of-frame overlapping reading frame in the ataxin-1 coding sequence encodes a novel ataxin-1 interacting protein. J Biol Chem. 2013;288(30):21824-35. doi: 10.1074/jbc.M113.472654.

5. Vanderperre B, Staskevicius AB, Tremblay G, McCoy M, O'Neill MA, Cashman NR, et al. An overlapping reading frame in the PRNP gene encodes a novel polypeptide distinct from the prion protein. FASEB J. 2011;25(7):2373-86. doi: 10.1096/fj.10-173815.

6. Klemke M, Kehlenbach RH, Huttner WB. Two overlapping reading frames in a single exon encode interacting proteins-a novel way of gene usage. EMBO J. 2001;20(14): 3849-60.

7. Abramowitz J, Grenet D, Birnbaumer M, Torres HN, Birnbaumer L. XLαs, the extra-long form of the α-subunit of the Gs G protein, is significantly longer than suspected, and so is its companion Alex. Proceedings of the National Academy of Sciences USA. 2004;101(22):8366-71.

8. Lee CF, Lai HL, Lee YC, Chien CL, Chern Y. The A2A adenosine receptor is a dual coding gene: a novel mechanism of gene usage and signal transduction. J Biol Chem. 2014;289(3):1257-70. doi: 10.1074/jbc.M113.509059.

9. Quelle DE, Zindy F, Ashmun RA, Sherr CJ. Alternative Reading Frames of the INK4a Tumor Suppressor Gene Encode Two Unrelated Proteins Capable of Inducing Cell Cycle Arrest. Cell. 1995;83:993-1000.
