# Supplementary material for: Overlapping genes and the proteins they encode differ significantly in their sequence composition from non-overlapping genes
Source: PLoS One. 2018 Oct 19;13(10):e0202513. doi: 10.1371/journal.pone.0202513 (PMC6195259; doi:10.1371/journal.pone.0202513)
Supplement: S4 Table — S4a Table shows the comparison of the pooled dataset of overlapping regions with that of the non-overlapping regions for 5 composition features. S4b Table lists the the 20 critical composition features peculiar to the overlapping gene dataset (chi-square > 100.0; 1 degree of freedom; P<0.00001; z score < -2.55; P <0.01). (DOC) [file pone.0202513.s005.doc]

**S4 Table. Details of the comparative analysis of overlapping and non-overlapping genes.**

S4a Table shows the comparison of the pooled dataset of overlapping regions with that of the non-overlapping regions for 5 composition features. S4b Table lists the the 20 critical composition features peculiar to the overlapping gene dataset (chi-square >100.0; 1 degree of freedom; P<0.00001, *z* score <-2.55; P<0.01).

**S4a Table.**

| Compositional feature | Chi-square | Degrees of freedom | P< |
| --- | --- | --- | --- |
| Nucleotides | 745.1 | 3 | 0.00001 |
| Dinucleotides | 1678.5 | 15 | 0.00001 |
| Amino acids | 1125.0 | 19 | 0.00001 |
| Amino acids (high, medium, or low codon degeneracy) | 360.9 | 2 | 0.00001 |
| Synonymous codons | 2242.2 | 58 | 0.00001 |

**S4b Table**

| Compositional feature | Percent content in the pooled overlapping dataset | Percent content in the pooled non-overlapping dataset | Percent difference (overlap – non-overlap) | Chi-square | z score (Wilcoxon test for paired data) |
| --- | --- | --- | --- | --- | --- |
| A | 27.03 | 29.97 | -2.94 | 136.4 | -2.56 |
| T | 22.24 | 25.95 | - 3.72 | 238.6 | -4.94 |
| C | 26.48 | 21.08 | 5.40 | 571.7 | -6.30 |
| AT | 5.80 | 7.81 | -2.01 | 187.7 | -5.19 |
| TA | 3.81 | 5.93 | -2.12 | 271.5 | -5.78 |
| TT | 5.42 | 7.15 | -1.72 | 128.0 | -4.85 |
| CG | 4.70 | 3.02 | 1.68 | 306.3 | -5.80 |
| CC | 7.35 | 4.85 | 2.50 | 431.6 | -4.30 |
| Arg | 7.47 | 5.27 | 2.19 | 188.0 | -5.62 |
| Ser | 9.51 | 7.27 | 2.24 | 146.5 | -5.60 |
| Pro | 7.31 | 4.98 | 2.33 | 221.7 | -3.90 |
| Tyr | 2.32 | 3.66 | -1.34 | 109.3 | -6.35 |
| Ile | 4.39 | 6.05 | -1.66 | 102.5 | -4.62 |
| High-degeneracy amino acids | 26.86 | 21.77 | 5.08 | 305.2 | -7.17 |
| Low-degeneracy amino acids | 36.62 | 41.59 | -4.97 | 209.5 | -6.09 |
| CGA (Arg) | 1.36 | 0.59 | 0.77 | 171.3 | -5.93 |
| TCG (Ser) | 1.26 | 0.61 | 0.64 | 116.8 | -5.45 |
| CCC (Pro) | 2.07 | 1.08 | 0.99 | 160.5 | -2.79 |
| CCG (Pro) | 1.37 | 0.65 | 0.71 | 135.1 | -4.42 |
| TAT (Tyr) | 1.05 | 2.18 | -1.13 | 124.9 | -5.38 |
